# Application of a Novel Measurement Setup for Characterization of Graphene Microelectrodes and a Comparative Study of Variables Influencing Charge Injection Limits of Implantable Microelectrodes

**DOI:** 10.3390/s19122725

**Published:** 2019-06-17

**Authors:** Ana Cisnal, Frank R. Ihmig, Juan-Carlos Fraile, Javier Pérez-Turiel, Víctor Muñoz-Martinez

**Affiliations:** 1 ITAP—Universidad de Valladolid, Paseo del Cauce 59, 47011 Valladolid, Spain; ana.cisnal@hotmail.com (A.C.); jcfraile@eii.uva.es (J.-C.F.); 2 Department of Biomedical Microsystems, Fraunhofer-Institut für Biomedizinische Technik (IBMT), 66280 Sulzbach/Saar, Germany; frank.ihmig@ibmt.fraunhofer.de; 3 Escuela de Ingenierías Industriales, Universidad de Málaga, Doctor Ortiz Ramos s/n, 29071 Málaga, Spain; vfmm@uma.es

**Keywords:** graphene microelectrode analysis, high frequency stimulation, cyclic voltammetry, voltage transient measurements, charge injection capacity

## Abstract

Depending on their use, electrodes must have a certain size and design so as not to compromise their electrical characteristics. It is fundamental to be aware of all dependences on external factors that vary the electrochemical characteristics of the electrodes. When using implantable electrodes, the maximum charge injection capacity (CIC) is the total amount of charge that can be injected into the tissue in a reversible way. It is fundamental to know the relations between the characteristics of the microelectrode itself and its maximum CIC in order to develop microelectrodes that will be used in biomedical applications. CIC is a very complex measure that depends on many factors: material, size (geometric and effectiveness area), and shape of the implantable microelectrode and long-term behavior, composition, and temperature of the electrolyte. In this paper, our previously proposed measurement setup and automated calculation method are used to characterize a graphene microelectrode and to measure the behavior of a set of microelectrodes that have been developed in the Fraunhofer Institute for Biomedical Engineering (IBMT) labs. We provide an electrochemical evaluation of CIC for these microelectrodes by examining the role of the following variables: pulse width of the stimulation signal, electrode geometry and size, roughness factor, solution, and long-term behavior. We hope the results presented in this paper will be useful for future studies and for the manufacture of advanced implantable microelectrodes.

## 1. Introduction

The most common method of charge injection in the context of functional electrostimulation (FES) is known as the galvanostatic or current-controlled method [1], which is based on the use of two-phase balanced charge electric pulses that inject the same magnitude of anodic and cathodic charge, resulting in zero charge transfer in a stimulation pulse [2]. For physiological reasons, the first phase of stimulation is typically cathodic and is used to obtain the desired function. The second phase is anodic and is used to reverse the electrochemical processes that occurred in the first phase [3,4].

The electrodes can be excited with different current waveforms; the only requirement is to avoid exceeding the limit charge values by which the electrode or the tissue can be damaged. The three-electrodes set-up is necessary when potential measurements are required due to the fact that it adds a third electrode known as the reference electrode (RE) that is used as a reference potential point. The RE is able to maintain its potential constant over time because, ideally, the current does not flow through it. The most common RE is Ag/AgCl (Silver/Silver Chloride). When both electrode and electrolyte phases are put in contact with each other, a redistribution of charge appears until the electrochemical equilibrium is achieved

Functional electrostimulation (FES) is a technique used to treat dorsal spine injuries, sensorial problems, as well as neurological problems. Applications of functional electrostimulation include restoration of hand function for individuals with cervical level spinal cord injury (SCI) [5], restoration of vision to the blind by using an array of electrodes attached to the retina [6,7], treatment of hearing problems [8,9], and deep brain stimulation for neural problems including Parkinson’s disease [10], involuntary movements, and psychiatric diseases such as depression and obsessive compulsive disorder [11,12].

FES consists in applying an electric pulse train (where the current amplitude goes progressively up and down) to a muscle or nerve [13]. Electrodes are the interface between the external circuitry and the tissue, delivering a charge that stimulates the nerves connected to the muscles of interest [14]. It is recommended that the pulse width is set between 100 and 400 microseconds and the frequency equal to or more than 20 Hz without exceeding 120 Hz, avoiding muscle fatigue or depolarization of the tissue cells [15]. The current amplitude must be high enough to cause muscle contraction, but not excessive, to avoid causing damage to the electrode or tissue.

Some emerging biomedical applications require the development of smaller electrodes that must be highly selective. However, such microelectrodes are required to deliver charge densities that exceed the traditional damage threshold, and consequently, the charge density is close to the safe limits at clinically effective levels [16]. Microelectrodes are characterized by high electrical impedance and not enough charge injection capacity (CIC) for some therapeutic applications because of their small geometric surface area (GSA). For this reason, alloys or coatings with high surface roughness are commonly used to significantly increase the effective surface area (ESA) of the electrode and, therefore, the amount of injected charge [17]. The effects of high frequency current stimulation on the polarization of three-electrode materials from voltage transients (VT) measured during sinusoidal current stimulation are presented in [18].

Many authors have worked in the electrochemical evaluation of microelectrodes for FES. In [19], the role of electrode geometry in terms of perimeter-to-surface area (PSA) ratio and shape using custom microelectrodes is examined. Their results show that the electrode shape may play a more significant role in the charge injection capability of microelectrodes. The impact of electrochemical performance on electrodes with high PSA has also been well documented [20,21,22]. Chemical and physical characteristics of microelectrodes such as long-term stability, high charge injection capacity, and low impedance play a role in successful treatment [23]. An electrochemical surface modification method for PEDOT that uses a PSS coating of neural interfaces involving a controlled cyclic voltammetry (CV) process is shown in [24]. This method is beneficial for neural interfaces as it increases the charge injection capacity of the electrode without degrading the mechanical stability of the coating. 

The design of safe stimulation protocols requires knowledge of the maximum charge that an electrode can inject to ensure that all reactions that occur in the electrolyte are reversible. This parameter is known as the “maximum reversible CIC” and is determined by studying the electrochemical behavior of the electrode.

In [25], we proposed and implemented a method to calculate the access voltage of implantable microelectrodes that does not require prior knowledge of the overpotential terms or the electrolyte (or excitable tissue) resistance, which is advantageous for in vivo electrochemical characterization of microelectrodes. Also in [25], we developed a measurement setup and an automated calculation method to determine the CIC of implantable microelectrodes. 

In this paper, we validate the method and setup presented in [25] by applying it to the characterization of a graphene microelectrode and to the measurement of the behavior of a set of microelectrodes. All of the methods were developed by the Fraunhofer IBMT (Institute for Biomedical Engineering). An exhaustive study was carried out on the dependence of the CIC with some microelectrode variables, using a 50 Hz balanced symmetric biphasic excitation signal. We electrochemically examined the role of the following microelectrode variables: pulse width of the stimulation signal, electrode geometry and size, roughness factor, solution, and long-term behavior. The new electrodes, made of graphene, were also tested and achieved good results, which could lead to the potential use of graphene as an advanced material for the microfabrication of stimulation electrodes. 

## 2. Materials and Methods 

### 2.1. Electrodes

**Figure 1:**
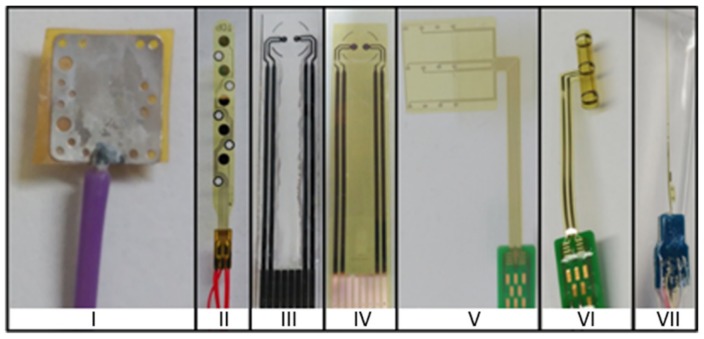
Microelectrodes designed and manufactured at Fraunhofer Institute for Biomedical Engineering (IBMT): (**I**) Pt foil, (**II**) Pt epimysial, (**III**) graphene printed, (**IV**) Pt printed, (**V**) Pt cuff, (**VI**) microporous Pt cuff, (**VII**) DS-File.

In this work, we applied a new method [25] to characterize different microelectrodes as listed in Table 1 and shown in Figure 1. All of the microelectrodes are suitable for neural stimulation and recording and have been designed and manufactured at Fraunhofer IBMT.

Epimysial electrodes are formed by five platinum contacts based on a 20 µm thick polyimide film with a diameter of 0.05 cm, 0.1 cm or 0.2 cm (electrodes B, C and D, respectively), and an inter-contact distance of 4 mm. They are used as recording electrodes, having the ability to measure small and deep muscles by recording the intramuscular electromyogram (iEMG). The electrodes are designed to be epimysially implanted; the electrode is placed underneath the epimysium—a sheath of fibrous elastic tissue surrounding a muscle [26].

**Figure 2:**
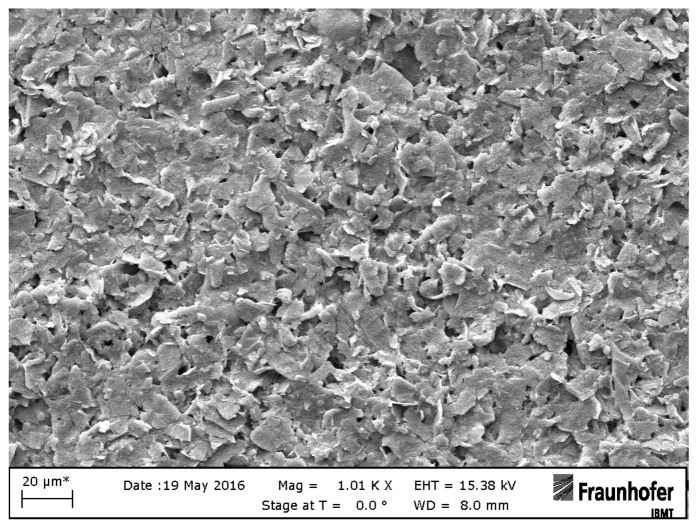
SEM image of a printed graphene electrode F with 20 µm resolution.

**Figure 3:**
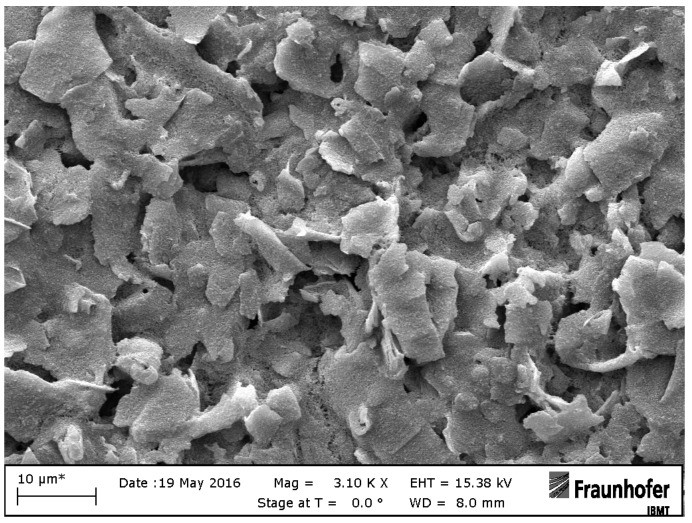
SEM image of a printed graphene electrode F with 10 µm resolution.

Printed electrodes have two round contacts with a diameter of 0.1 cm and are printed on a flexible polymer foil for impedimetric or electrochemical measurements. Graphene electrodes F are manufactured using the R2R gravure printing process using commercial graphene ink (HDPlas^®^ IGSC02002, Haydale Ltd., Ammanford, UK). HDPlas^®^ Graphene Nanoplatelets are applied to a sputtered platinum electrode E by means of a thin needle which is used as a kind of brush [27]. Graphene nanoplatelets, which are characterized by 10–100 layers 3–30 nm thick, have the same electrical properties as highly ordered pyrolytic graphite (HOPG) [28]. Figure 2 and Figure 3 show representative SEM images of printed graphene electrode F.

Cuff electrodes are suitable for recording and stimulating peripheral nerves. The cuff electrodes are implanted around the nerve, making selective neuromuscular activation possible. The nerve cuff electrodes are highly flexible and are made of polyimide with integrated platinum contacts with a 5 mm interelectrode distance. The planar electrode G is rolled to become a cylinder and fixed in the final cuff-shape (electrodes H and I). The whole structure is designed with physical properties and dimensions that avoid compression and stretch [29].

DS-File electrodes are a new generation of intramuscular multi-channel electrodes for electromyogram (EMG) recording and muscle stimulation. The electrode combines recording and stimulation contacts on a single thin polyimide filament. The electrode is equipped with twelve small recording contacts (electrode K) on one side of the structure and three large stimulation contacts (electrode J) on the other side. The electrode is created using a double-side process, and the electrode contacts are coated to reduce the impedance and to increase CIC [30].

### 2.2. Measurement of the Charge Injection Capacity. Experimental Setup

Voltage transient (VT) measurements were performed by applying a current-controlled stimulation pulse to a working electrode (WE) while its potential with respect to the reference electrode (RE) was recorded. For the VT technique, we used the EasyStim pulse stimulator developed at Fraunhofer IBMT, which provides a charge-balanced biphasic symmetric waveform. A cathodic-first excitation waveform was used, and its frequency was set to 50 Hz to work with the most common parameters used in biomedical applications. We recorded the potential transients of the WE with respect to the RE. The magnitude of the current actually applied was also controlled.

**Figure 4:**
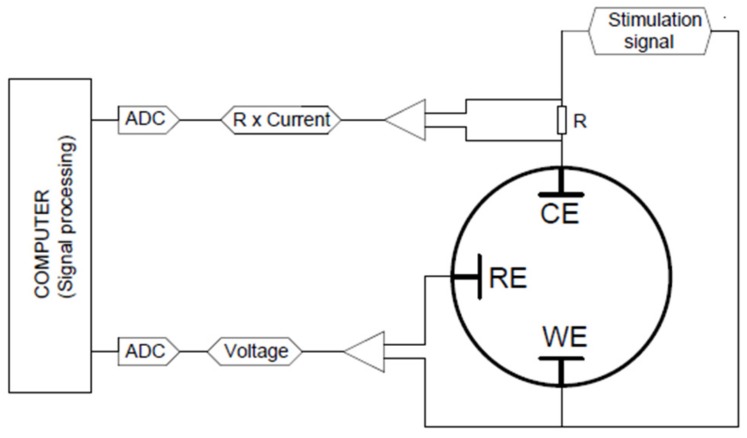
Simplified diagram of the voltage transient (VT) measurement setup used in our lab. WE, RE, and CE represent the working, reference, and counter electrodes, respectively.

Figure 4 shows a simplified diagram of the setup developed to make VT measurements for characterization of microelectrodes. 

To determine the CIC, we analyzed the potential transient on the WE using a specially developed program based on the OriginPro platform (OriginLab Corp., Northampton, MA, USA). More details about the custom circuit board (shown in Figure 4) and the signal analysis are outlined in [25].

With the setup shown in Figure 4, a stimulation signal was applied to the WE. The VTs on the WE with respect to the RE were recorded and imported into the computer. These signals were processed to calculate the access voltage, the extreme polarization potentials, and the CIC. The processing was done in OriginPro 2015 (OriginLab Corp., Northampton, MA, USA) using the Origin C programming language. 

The VWe−Ret signal (the WE potential with respect to the RE) was filtered to smooth it out and remove random noise by using a Savitzky–Golay filter (five points, second order). The offset and the derivation were calculated from the filtered signal. Next, the times at which the stimulation pulse changes sign, the pulse width, and the access voltage were determined from the derivation.

The $I_{\mathrm{We}-\mathrm{Ce}}\left(t\right)$ signal (the current from the WE to the counter electrodes (CE), which is the stimulation current in mA) was filtered by a Savitzky–Golay filter (30 points, second order) to calculate the amplitude of the stimulation current.

### 2.3. Measurement of the Cathodal-Storage Capacity. Experimental Setup

Cyclic voltammetry (CV) techniques allow the type and magnitude of electrolyte reactions to be analyzed and the electrochemical potential window to be determined. CV measurements were made by using commercially available interconnected modules from Solartron Analytical, UK: 1260A. Five cycles were executed to ensure that the electrode reached its steady state. These five cycles were recorded at a sweep rate of 0.1 V/s, beginning at the open circuit potential and sweeping first in the positive direction. 

The cathodal charge-storage capacity is the total amount of available reversible charge in a cathodic phase of a pulse of stimulation per area. That means that the current is the cathodic current, the limits of integration are the limits of the water window (the potential region within the charge transfer is reversible). So, cathodal charge-storage capacity expresses a surface density charge and is the total net charge amount. 

To calculate cathodal charge-storage capacity, the following formula was used [31]:

(1) $${\mathrm{CSC}}_{c}=\frac{1}{v}|\int_{Ec}^{Ea}j_{c}\left(u\right)dU|,$$

where CSC_c_ is the cathodal charge-storage capacity $\left(mC/cm^{2}\right)$, v is the sweep rate (mV/s), $j_{c}\left(u\right)$ is the cathodal current density $(mA/cm^{2}$), $E_{c}$ and $E_{a}$ are the cathodic and anodic extremes of the water window respectively, and U is the potential with respect to the RE (V). The calculation of tge cathodal charge-storage capacity was implemented in the above-mentioned software in OringPro.

The CV and VT techniques require the configuration of three electrodes. We used an Ag/AgCl-type reference system (B2920+) and a large-area Pt auxiliary electrode (Pt 1800) from the BlueLine family of Schott Instruments, Germany. The electrolyte was a room-temperature saline isotonic solution (NaCl, 0.145 M). 

## 3. Results and Discussion

This section describes and discusses the results of the measurements performed with the microelectrodes listed in Table 1 using our measurement setup and our automated calculation method, both described in more detail in [25]. The charge injection capacity of microelectrodes was determined using the homemade electronics board presented in [25]. Automated calculations were done by using the program that we developed on the ORIGIN platform. This program allows for the performance of a comparative study of different electrodes immersed in an isotonic solution as the electrolyte (unless otherwise indicated) at constant room temperature based on the pulse width of a 50 Hz symmetric biphasic excitation signal. 

### 3.1. Analysis of Variables That Influence the Characterization of Electrodes

The electrochemical process which occurs at the electrode–electrolyte interface is strongly influenced by the characteristics of the stimulation signal. It has been reported that shape and size of electrodes has a significant impact on the breakdown mechanism of any dielectric [32], and the effect of electrode location has also been studied [33]. Long term stability is also an important aspect for electrodes for functional electrostimulation because electrodes are interfaced with tissues over long time periods in the case of a medical implants. To evaluate the stability, we performed a long-term characterization of microelectrode electrical properties for a period of 7 days.

Three electrode properties that allow electrochemical characterization are reversible charge injection capacity, cathodal charge-storage capacity, and impedance of the electrode–electrolyte interface over a wide range of frequencies. The values of these characteristics were determined for the microelectrodes shown in Figure 1 by examining the role of the following microelectrode variables: pulse width of the stimulation signal, electrode geometry and size, roughness factor, distance to the reference electrode, solution, and long-term behavior. 

#### 3.1.1. Pulse Width of the Stimulation Signal 

**Figure 5:**
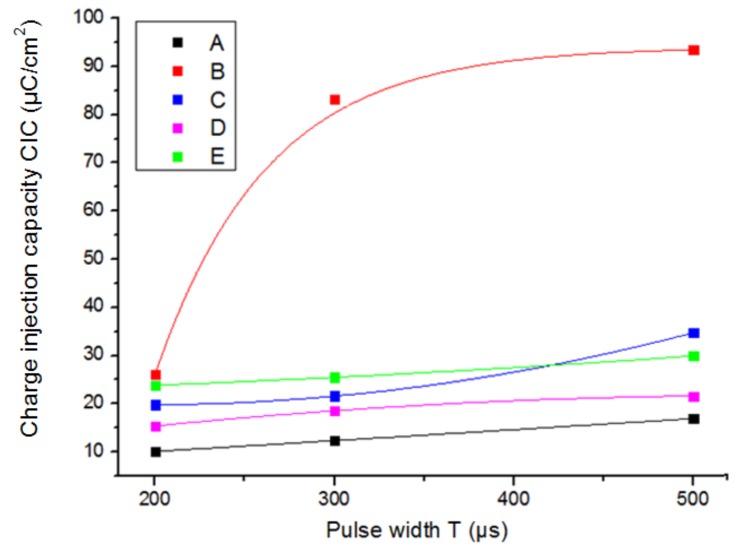
Maximum reversible charge injection capacity (CIC) as a function of the pulse width of the stimulation signal.

A study was performed to observe the variation of the maximum reversible CIC of an electrode with the pulse width of the stimulation signal, and how this variation was also related with the size of the electrode. With this aim, five electrodes (A, B, C, D, and E, Table 1) of the same material (platinum) and different geometric area were chosen to be the subjects of the study. For each electrode, the CIC was measured when applying a 50 Hz stimulation signal with different pulse widths (200, 300, 400, and 500 μs). The results are shown in Figure 5. 

Lower values of CIC were obtained with shorter pulse widths of the stimulation signal, because the central region of the electrode was not used due to the non-uniform current distribution. With longer pulses, the reactions started to spread all over the surface of the electrode and higher charge injection capacities were observed. The trend of incremental CIC changes with the pulse width for the same kind of electrodes (epimysial electrodes B, C, D) was higher for smaller geometric surface areas (GSA of electrode B was smaller than C, which was smaller than D).

**Figure 6:**
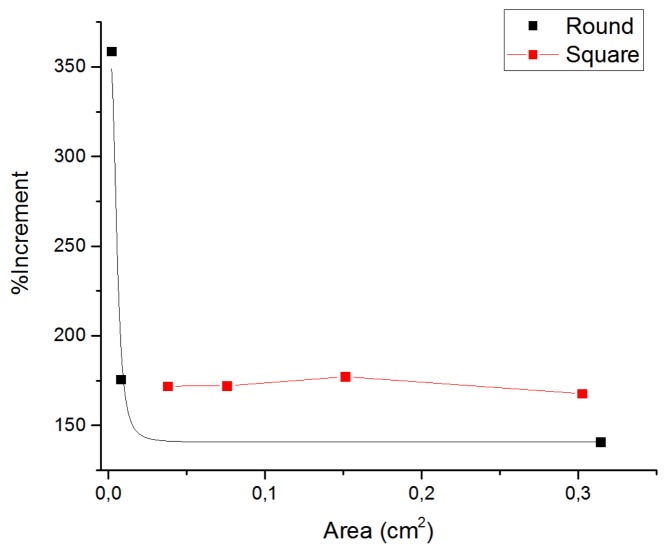
Incremental changes of the CIC when round electrode dimensions become smaller. Black line: round epimysial electrodes B, C, D; Red line: Pt foil square electrode A.

For round electrodes, the incremental changes of the CIC with longer pulses became higher as the electrode dimensions became smaller, as shown in Figure 6, where the percentage increase was calculated as the quotient of the CIC measured for 500 and 200 μs pulse widths.

#### 3.1.2. Geometry Surface Area (GSA) of the Electrode

**Figure 7:**
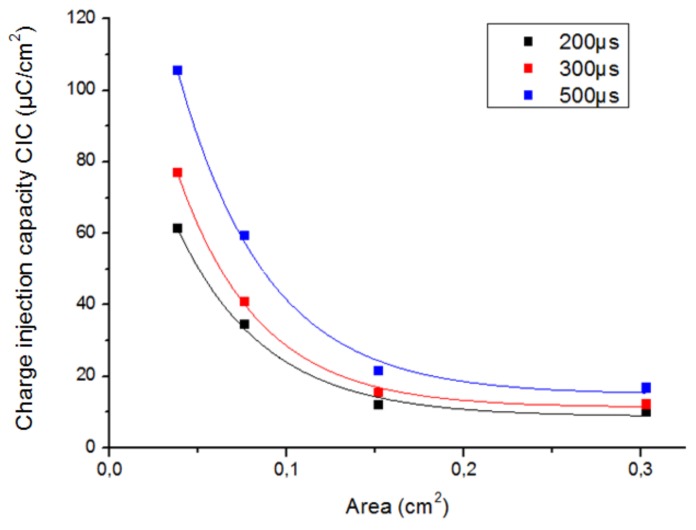
CIC of the foil Pt square electrodes A as a function of the electrode area for three different pulse widths of the stimulation signal.

**Figure 8:**
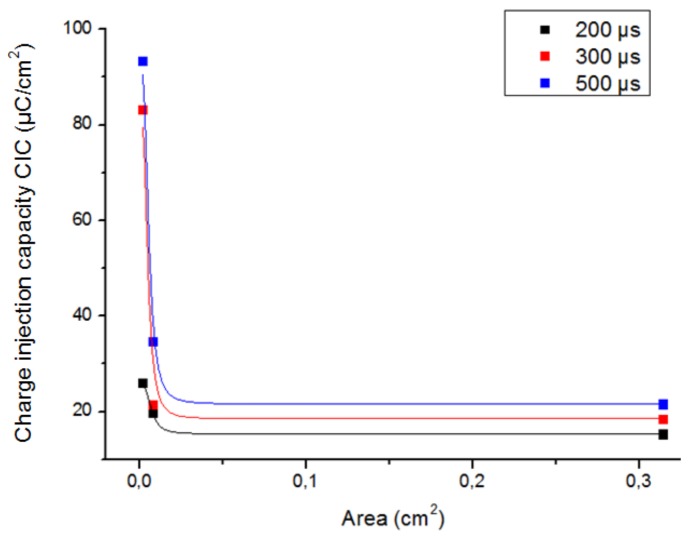
CIC of the epimysial sputtered Pt round electrodes B, C and D as a function of the electrode area for three different pulse widths of the stimulation signal.

The size and shape of an electrode influence the CIC because of the non-uniform current distribution, which implies that the reactions occur on the border region of the electrode. The influence of GSA of an electrode on its maximum reversible CIC was observed for three different pulse widths of the stimulation signal in two different electrodes: foil platinum square electrodes (Figure 7) and epimysial sputtered platinum round electrodes (Figure 8).

If the GSA of the electrode decreases, the mass transport rate also decreases with the result that the irreversible reactions take longer to occur and the electrode experiences an increase in the CIC.

It can be observed in Figure 7 and Figure 8 that the behavior between the CIC and the electrode area is non-linear, and it is different depending on the shape of the electrode. Higher values for delivered charge density were obtained using round electrodes than using square shape ones. For instance, although the square electrode A was slightly smaller and had a roughness factor higher than one, it was characterized by a smaller value of CIC than the round electrode D; both electrodes were made of platinum (see Table 2).

To summarize, our results show that it is preferable to use round electrodes with small areas in order to achieve high values of delivered charge density.

#### 3.1.3. Roughness Factor of the Electrode

**Figure 9:**
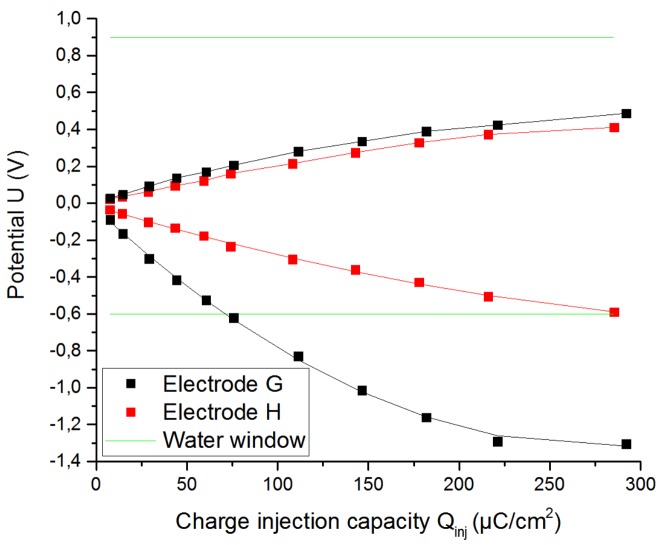
Increase of the delivered charge because of a higher roughness factor for a pulse width of 200 µs.

**Figure 10:**
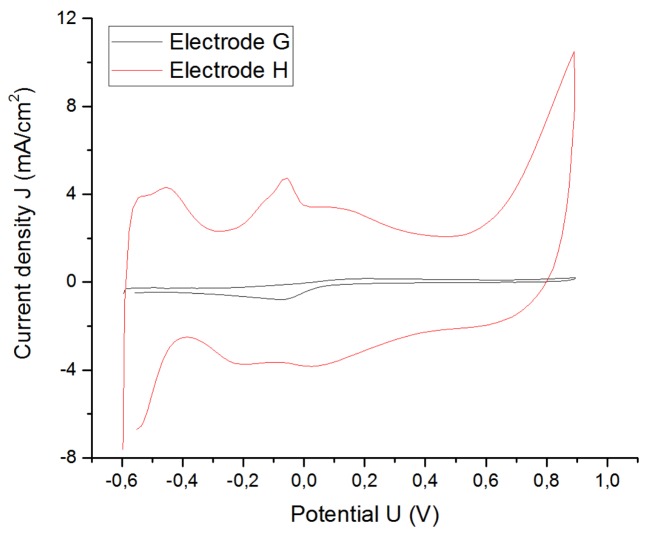
Increase of the CSC_c_ because of a higher roughness factor.

**Figure 11:**
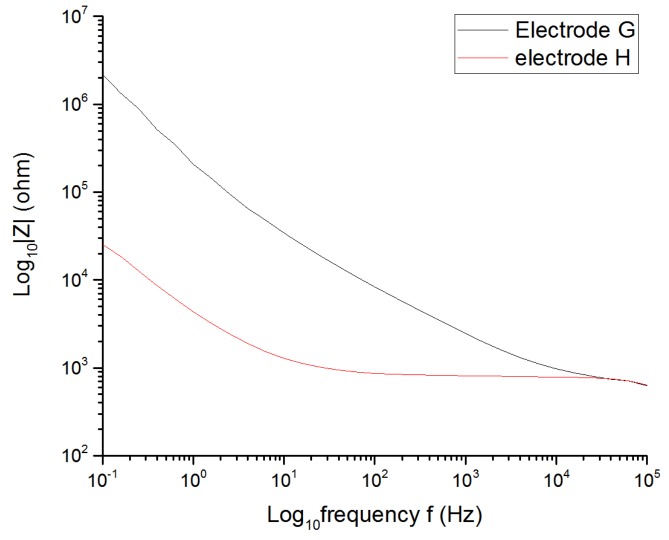
Decrease of the low frequency electrode impedance because of a higher roughness factor.

The purpose of coating the electrode is to decrease the impedance and achieve higher values of CIC. That means that with higher roughness factors (GSA/ESA ratio), higher delivered charge densities are available. This fact can be observed in Figure 9, Figure 10 and Figure 11 that show the results from when two electrodes G, H with the same shape, size, type, and material were tested by applying the same stimulation signal. The electrode G was the electrode without coating and electrode H was the coated electrode. Table 3 summarizes all the data obtained from the Figure 9, Figure 10 and Figure 11.

The CIC and the CSC_C_ of the microporous electrode increased by 362% and 1019%, respectively, with respect to the uncoated electrode due to the significant decrease in its impedance as well as the resistance of the solution (from 884.8 to 770 Ω).

#### 3.1.4. Influence of Electrolyte Composition

Another interesting aspect that was studied was the behavior of the electrodes immersed in different solutions. Pt cuff electrode G was placed in different solutions, such a NaCl solution and PBS (phosphate-buffered saline), at room temperature. The formulation of both solutions is shown in Table 4.

Both solutions have NaCl as their main component in similar concentrations and, therefore, their resistivity is quite similar. The main difference between the solutions is that PBS is a buffer solution, whilst the isotonic saline solution is an unbuffered one. A buffer is a solution that maintains its pH despite additions of acid or base over a range, whereas an unbuffered solution is one whose pH is not stabilized. For this reason, PBS is closer to in vivo conditions than is the NaCl solution.

**Figure 12:**
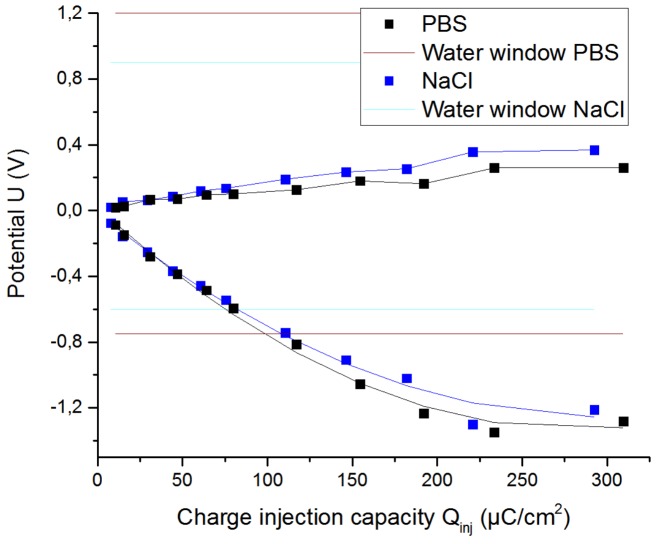
Extreme polarization for the Pt cuff electrode G in PBS and NaCl solutions, using a stimulation signal of 200 µs.

**Figure 13:**
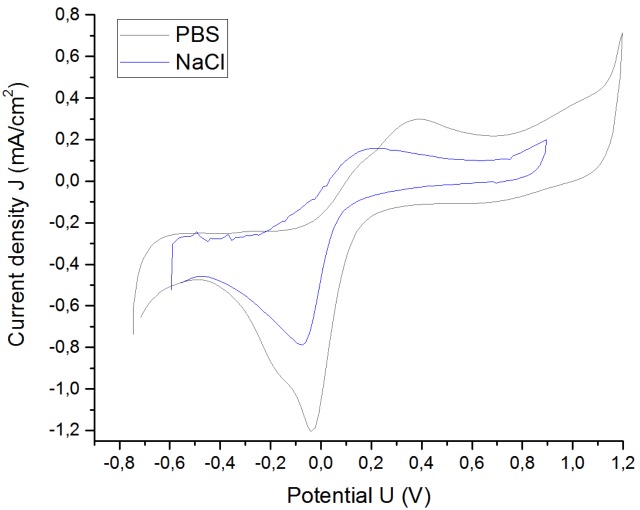
Cyclic voltammetry (CV) of the Pt cuff electrode G in PBS and NaCl solutions.

The VT was performed with a pulse width of 200 µs (see Figure 12). The CV was performed to determine the water window for platinum in a PBS solution (see Figure 13). 

**Figure 14:**
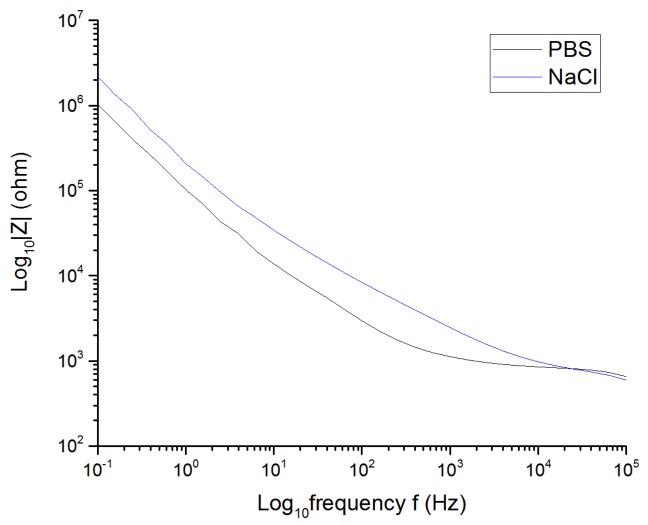
Impedance of the Pt cuff electrode G in PBS and NaCl solutions.

The low frequency impedance decreased, and the water window increased with increasing buffer, because there were more H+ and OH− counterions available and, as a result, more reversible delivered charge was possible (see Figure 14). The electrical impedance spectroscopy response at high frequencies was similar, due to the fact that NaCl and PBS solutions have similar conductivities. Table 5 shows the results.

The CIC, the cathodal charge-storage capacity, and the potential range related to the water window rose considerably using PBS as the solution instead of NaCl. 

#### 3.1.5. Long-Term Behavior

The Pt cuff electrode G was placed in a solution of NaCl at room temperature for 172 h (around 7 days) to study the changes that this electrode may experience during a continuous pulse test. A charge-balanced biphasic symmetric stimulation pulse was applied for the duration of the experiment. The pulse width was set to 200 µs and the amplitude of the current was 0.3 mA. This ensured that the reactions that occurred would always be reversible, and there would be no damage in the electrode or changes in the electrochemical composition of the solution. 

**Figure 15:**
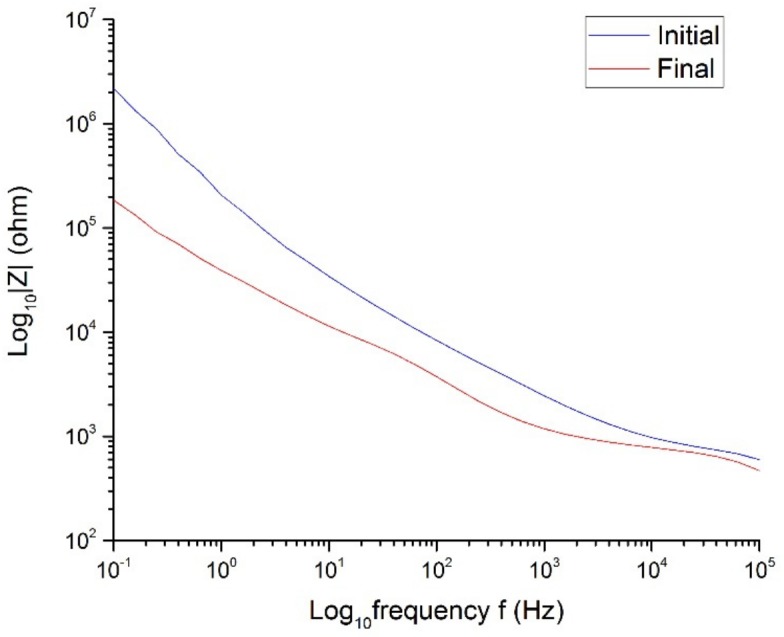
Impedance of the Pt cuff electrode G in NaCl at room temperature.

**Figure 16:**
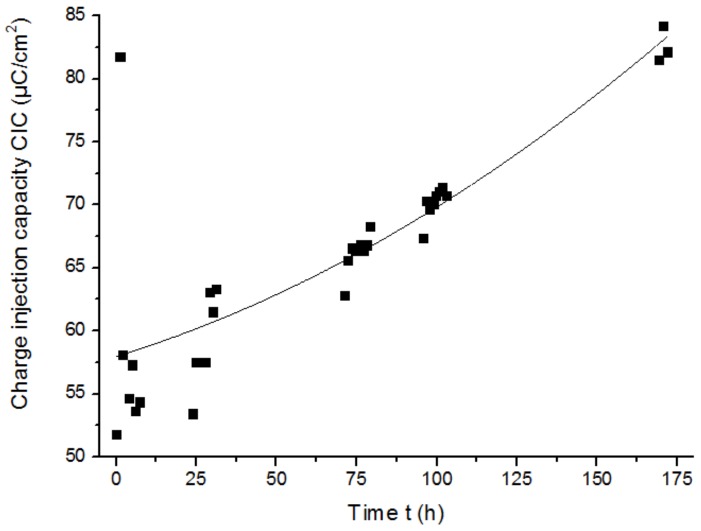
CIC of the Pt cuff electrode G over time.

**Figure 17:**
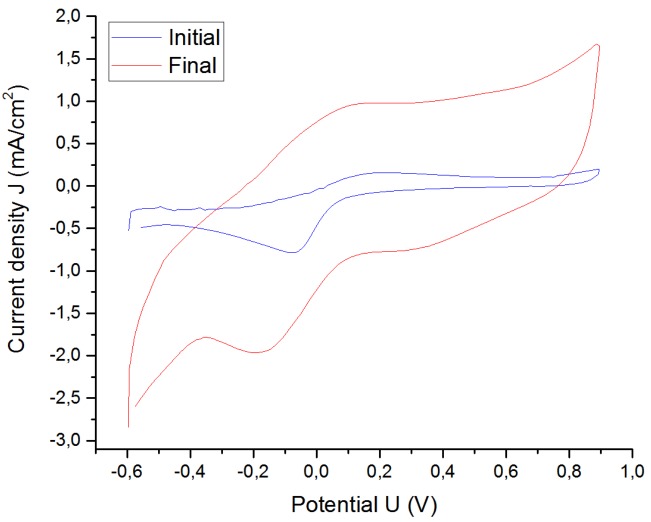
CV of the Pt cuff electrode G in NaCl at room temperature.

The absolute impedance at low frequencies, which represents the impedance of the electrode, was smaller after one week (see Figure 15), which resulted in a significant increase of the CIC (see Figure 16) and the ${\mathrm{CSC}}_{c}$ (see Figure 17). The explanation for this is that the electrolyte was penetrating the electrode contacts over the experiment time, increasing the effective area [17].

On the other hand, the high-frequency impedance decreased after 172 h of stimulation (see Figure 15). Significant impedance reduction over time was experienced for carbon polymer pastes previously [34]. This phenomenon might be explained because the increase of effective area of the electrode due to its rough nature could result in a drop of impedance. Table 6 summarizes the results obtained from the Figure 15, Figure 16 and Figure 17.

### 3.2. Characterization of Graphene Microelectrodes

We have presented the results we obtained by electrochemical evaluation of the CIC of the microelectrodes by examining the role of their main variables. Next, we apply our measurement setup and automated calculation method to characterize graphene microelectrodes.

Graphene is an allotrope of carbon. It is formed by carbon atoms shaped as a hexagonal lattice made of covalent bonds of carbon atoms. The carbon in the two-dimensional structure of one atom of thickness has many unusual and extraordinary properties. Graphene is characterized by a high conductivity: the charge mobility can be higher than 15,000 cm^2^ V^-1^ s^-1^ under ambient conditions [35]. Graphene provides excellent electrochemical properties, high surface area, mechanical strength, high flexibility, and biocompatibility, and thus is ideal for electrode fabrication [36,37]. 

**Figure 18:**
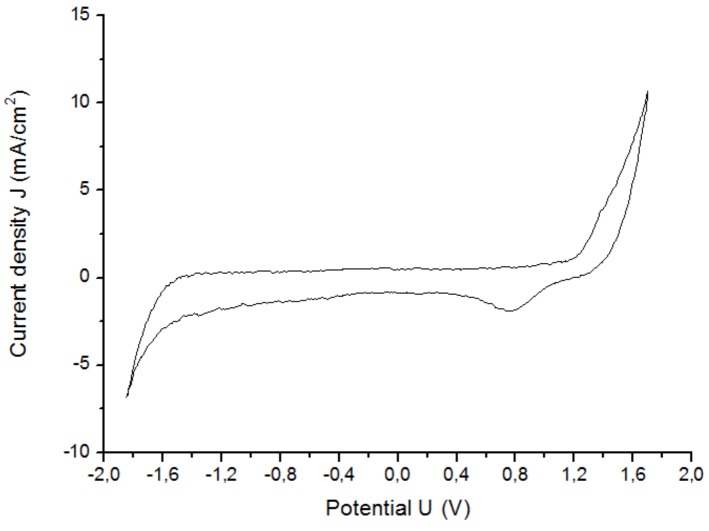
CV of the graphene electrode F in NaCl solution.(scan rate = 100 mV, scan number = 5).

The CV was performed with the graphene printed electrode F in the NaCl solution to determine the water window (Figure 18). The potential region where there were no irreversible reactions in the electrolyte for the graphene was [−1.85, 1.7] V, which is quite a lot larger in comparison with platinum [−0.6, 0.9] V. Although no values for the water window of graphene in NaCl could be found in the literature, the results for other carbon allotropes, such as glassy carbon [−0.64, 1.66] V and HOPG [−0.72, 1.94] V, verify the wide electrochemical window for the graphene [38]. 

Graphene’s large inert potential window makes it ideal to be used in biomedical applications as an electrode. Higher charge injection capacities are possible than with Pt electrodes, because of the significant increment of the water window.

However, achieving a large CIC for graphene electrodes requires a previous electrochemical activation of the material itself. The electrochemical activation of graphene electrodes is accomplished by applying multiple sweep-rate potentials between the water window limits, allowing emerging electrochemical reactions in the material to change its behavior [39,40]. The activation causes the fracture of the graphene lattice, creating more pores and thus increasing the edge plane density with a result of an increase in the electrode-transfer rate and the charge-injection capacity [41].

**Figure 19:**
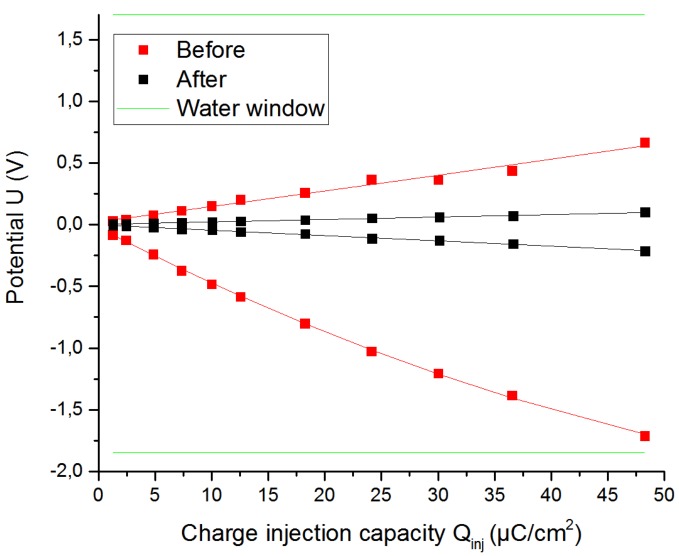
Extreme polarization potentials for the graphene electrode F before and after the activation using a stimulation signal of 200 µs.

Figure 19 shows the extreme polarization potential of the flexible printed graphene electrode F before and after the activation.

**Figure 20:**
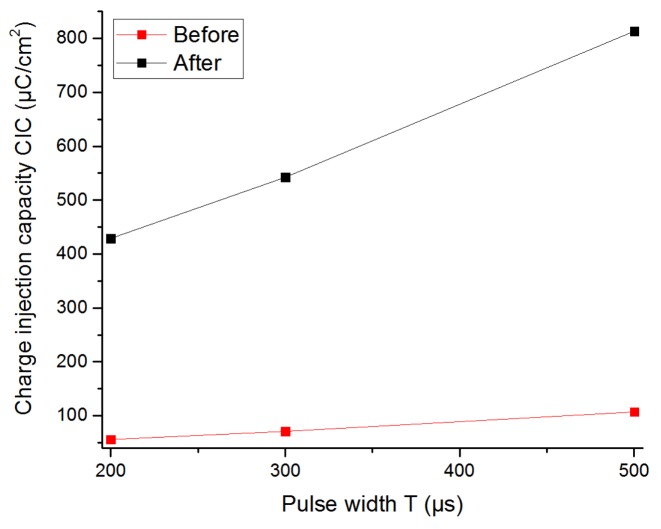
CIC of the graphene electrode F as a function of the pulse width of the stimulation signal. Red line: before the activation; Black line: after the activation.

Figure 20 shows the CIC of the flexible printed graphene electrode F before and after the activation. The values obtained from Figure 20 are summarized in Table 7, and they are consistent with [42], where CIC was found to be 500 µC/cm^2^ for a 200 µs pulse. They indicate that the CIC of the graphene electrode F after activation increased around 6–7 times, because the electrolyte resistance of the graphene decreased by a factor of two with the activation (from 360 Ω to 165 Ω). However, CIC became significantly smaller after five weeks of the activation, as seen with the results in Table 7.

As we can see in Figure 1, electrodes III and IV have identical geometry. The electrode III is a graphene printed electrode F, and electrode IV is a Pt printed electrode E. Electrode E was built using a flexible printed electrode made of platinum as a base, and then, the graphene was bonded to the electrode contact, and it was heated up to 60 °C for 30 min to achieve the correct adhesion. A comparative study of both electrodes E and F was carried out. Table 7 shows the CIC values obtained for the Pt printed electrode E and the graphene printed electrode F. 

A detachment of graphene in the contact of the electrode E after performing these tests was found. The poor adhesion between the graphene used as a coating material and the metal electrode during the stimulation was reported in previous studies [43,44].

### 3.3. Comparison between CIC and CSC_c_

Table 8 summarizes the results of the CIC obtained in Section 3.1 and Section 3.2. It shows the CIC and cathodal charge-storage capacity for all the electrodes shown in Table 1, sorted by decreasing values of CIC with the aim of visualizing the influence of the electrode characteristics. The CIC was calculated through the VT performed with a 200 µs pulse width, whilst the CSC_c_ was obtained by the CV with a scan rate of 100 mV/s.

The highest CIC was found for the graphene printed electrode F. A high amount of reversibly-delivered charge availability was also determined for coated electrodes made of Pt with small dimensions (electrodes H and K). The lowest values were found for square electrodes A and sputtered Pt electrodes with large dimensions (electrodes C and D).

## 4. Conclusions

An exhaustive study about the dependence of the CIC with some variables for a balanced symmetric biphasic excitation signal of 50 Hz was carried out with the microelectrodes listed in Table 1. The CIC dependence on some variables was observed in previous studies and the behavior that was found matched what was expected.

Knowledge of the CIC is vital to design safe stimulation protocols; it provides information on the effectiveness of the stimulation, and allows for the determination of the maximum current amplitude of the stimulation signal that can be applied to the electrode without damaging the electrode or the target tissue. The characterization of the set of eleven electrodes (Table 1) made in this paper, especially the calculation of CIC and the cathodal charge-storage capacity, was performed automatically using home-made software that calculates the CIC of the electrode through the derivative of the voltage transients, using the previous knowledge of the electrochemical water windows and the size of the electrode.

We verified that area and geometry as well as pulse width of stimulation signal have a strong influence in voltage-transient measurement, because the reactions do not occur uniformly across the electrode area. Larger reversible charges were possible with electrodes whose effective surface area were higher than their geometric surface area. The Pt cuff electrode G was studied for seven days, resulting in an increase of the reversible CIC and a non-stable long-term electrode, which is a common issue in electrodes [34]. 

The new generation of electrodes made of print graphene were tested and achieved good results; this may lead to the potential use of graphene as an advanced material for the microfabrication of stimulation electrodes. The amount of reversible charge that can be injected with graphene electrodes is considerably larger than that of other electrodes with similar sizes and shapes.

In summary, the characteristics of an electrode depend on a large number of factors. For this reason, the total characterization of an electrode includes the study of the behavior of the electrode as a function of all the variables analyzed in this paper.

## Figures and Tables

**Table 1:** Microelectrodes characterized in the present study. GSA = geometric surface area.

Microelectrode	Type	Material	Shape	GSA (cm^2^)
A	Foil	Pt	Square	0.3025
B	Epimysial	Sputtered Pt	Round	0.001963
C	Epimysial	Sputtered Pt	Round	0.007854
D	Epimysial	Sputtered Pt	Round	0.031416
E	Print	Sputtered Pt	Round	0.007854
F	Print	Graphene	Round	0.007854
G	Cuff	Sputtered Pt	Ellipse	0.0013
H	Cuff	Microporous Pt	Ellipse	0.0013
I	Cuff	Microporous Pt	Rectangular	0.0195
J	DS-File	Microporous Pt	Round	0.004
K	DS-File	Microporous Pt	Round	0.00006963742

**Table 2:** Comparison of the CIC of electrodes A and D with similar dimensions, but different roughness factors.

Microelectrodes	Roughness Factor	GSA (cm^2^)	CIC 200 µs (µC/cm^2^)	CIC 300 µs (µC/cm^2^)	CIC 500 µs (µC/cm^2^)
A	>1	0.3025	10.08	12.33	16.91
D	1	0.3114	15.37	18.54	21.61

**Table 3:** Characteristic values of Pt cuff electrodes G and H with the same shape and size, but different roughness factors. ESA = effective surface area; CSC_c_ = cathodal charge-storage capacity.

Electrode	Material	GSA/ESA	CIC (µC/cm^2^)	CSC_c_ (mC/cm^2^)	Rs (Ω)
G	Pt	1	81.63	3.69532	884.8
H	Microporous Pt	>1	295.9	37.6721	770

**Table 4:** Formulation of phosphate-buffered saline (PBS) and NaCl solutions.

	PBS	NaCl
NaCl	8000	9000
KCl	200	-
${\mathrm{KH}}_{2}{\mathrm{PO}}_{4}$	200	-
${\mathrm{Na}}_{2}{\mathrm{HPO}}_{4}-7H_{2}O$	2160	-

**Table 5:** Values of the cuff electrode G in PBS and NaCl solutions.

Solution	CIC (µC/cm^2^)	CSC_c_ (mC/cm^2^)	Water Window (V)
**NaCl**	81,63	3,695	[−0.6, 0.9]
**PBS**	97,76	6,409	[−0.75, 1.2]

**Table 6:** Results at the beginning and end of the continuous pulse test for the cuff electrode G.

	CIC (µC/cm^2^)	CSC_c_ (mC/cm^2^)
Initial (t = 0 hours)	57.97	3.695
Final (t = 172 hours)	83.38	14.86

**Table 7:** CIC of the graphene printed electrode F and Pt printed electrode E for different values of pulse width.

	CIC 200µs (µC/cm^2^)	CIC 300µs (µC/cm^2^)	CIC 500µs (µC/cm^2^)
Graphene electrode F before activation	55.9	71.05	106.84
Graphene electrode F after activation	428.9	542.3	813.6
Graphene electrode F 5 weeks after activation	145.7	177.3	242.8
Pt printed electrode E	23.83	25.48	29.92

**Table 8:** CIC and CSC_c_ of all electrodes of our study, sorted by decreasing CIC.

Microelectrode	Type	Material	Area (cm^2^)	CIC (µC/cm^2^)	CSC_c_ (mC/cm^2^)
F	Print	Graphene	0.007854	428.9	31.2806
H	Cuff	Microporous Pt	0.0013	295	37.6721
K	DS-File	Microporous Pt	0.00006963742	231.2	9.88699
I	Cuff	Microporous Pt	0.0195	166.9	29.6721
J	DS-File	Microporous Pt	0.004	122.7	15.4088
G	Cuff	Sputtered Pt	0.0013	81.63	3.69532
B	Epimysial	Sputtered Pt	0.001963	26.06	6.26489
E	Print	Sputtered Pt	0.007854	23.68	3.19265
C	Epimysial	Sputtered Pt	0.007854	19.78	2.42284
D	Epimysial	Sputtered Pt	0.031416	15.37	0.211339
A	Foil	Pt	0.3025	10.08	1.8863
